# Screening for colorectal cancer: A recommendation statement of the Austrian National Committee for Cancer Screening

**DOI:** 10.1007/s00508-023-02209-0

**Published:** 2023-05-31

**Authors:** Gerald Gartlehner, Eva Schernhammer, Sigurd F. Lax, Matthias Preusser, Herbert Bachler, Harald Titzer, Maria Kletecka-Pulker, Helga Turnher, Uwe Siebert

**Affiliations:** 1 Department for Evidence-based Medicine and Evaluation, University of Krems, Dr.-Karl-Dorrek-Str. 30, 3500 Krems, Austria; 2 RTI International, Research Triangle Park, NC USA; 3 Center for Public Health, Department of Epidemiology, Medical University of Vienna, Kinderspitalgasse 15, 1090 Vienna, Austria; 4 Pathology, Hospital Graz II, Graz, Austria; 5 Johannes Kepler University, Linz, Austria; 6 Division of Oncology, Department of Medicine I, Medical University of Vienna, Vienna, Austria; 7 Tyrolean College of General Medicine, Medical University of Innsbruck, Innsbruck, Austria; 8 Austrian Society of Hematology and Oncology Nurses, Vienna, Austria; 9 Institute for Ethics and Law in Medicine, University of Vienna, Vienna, Austria; 10 Colorectal Cancer Patient Support Group, Vienna, Austria; 11 UMIT TIROL—University for Health Sciences and Technology, Hall in Tirol, Austria; 12 Harvard T.H. Chan School of Public Health, Boston, USA; 13 Institute for Technology Assessment, Massachusetts General Hospital, Harvard Medical School, Boston, MA USA

**Keywords:** Prevention, Guideline, Colonoscopy, Fecal occult blood test, Nationwide, Austria

## Abstract

**Background:**

Colorectal cancer is the fourth most common cancer in Austria. To date, colorectal cancer screening in Austria remains opportunistic and includes colonoscopy or stool-based blood tests. The Austrian National Committee for Cancer Screening developed evidence-based recommendations for a nationwide organized colorectal cancer screening program.

**Methods:**

The methodological framework followed the approach of the United States Preventive Services Task Force. The evidence base underlying the newly developed recommendations comprised a review of the existing published evidence and a decision analytic model tailored to the Austrian context. Using a structured process, committee members considered 1) the magnitude of the net benefit of each screening strategy, 2) the certainty of evidence, and 3) the level of acceptance of the interventions among the target population.

**Recommendations:**

The Austrian National Committee for Cancer Screening recommends the implementation of a nationwide organized colorectal cancer screening program for all adults aged 45–75 years. For persons 65 years or older, screening decisions should occur on an individual basis in accordance with a person’s overall health, prior screening history, and preferences.

Specifically, the committee recommends either a 10-year screening colonoscopy or biennial fecal immunochemical tests with colonoscopy following a positive result, with both screening strategies considered equivalent. Each citizen should be able to make an informed decision about their preferred screening method. Switching between the two screening strategies should be possible. Following an unremarkable colonoscopy, screening by fecal immunochemical test (FIT) is only required after 10 years. Screening recommendations apply only to asymptomatic persons at average risk for colorectal cancer.

The screening program must be pilot tested, and accompanied by a public information campaign, formative evaluation, quality assurance, and data collection.

## Introduction

### Colorectal cancer burden in Austria

Colorectal cancer is the fourth most common cancer in Austria [1] overall (third most common for men and women separately) [2, p. 55] and the second most common cause of overall cancer deaths (third most common for men and women separately). More men (57.1% of all new cases) than women (42.9%) are affected by colorectal cancer. Between 2017 and 2019, approximately 60% of all colorectal cancer diagnoses in Austria were made after the tumor had already penetrated the organ boundaries (localized stage 40.1%; regionalized stage 43.7%; disseminated stage 16.2%). In Austria, the colorectal cancer incidence rates decreased from 1983 to 2016, with similar decreases from 2003 to 2016 for all 5‑year age groups 45 years or older. While colorectal cancer is exceedingly rare among younger individuals, an increase in incidence was observed in the Austrian population younger than 35 years. Furthermore, the colon cancer incidence rates appear to increase in men and women aged 45–49 years (just outside the currently recommended screening age range), but not in 50–54-year-olds. Although based on small numbers, this increase in incidence rates among younger adults is consistent with that observed in other European countries. Among the risk factors for colorectal cancer that have been attributed to the recent rise of colorectal cancer in the younger age groups are obesity, an unhealthy (processed meat-based) diet, smoking, excess of alcohol consumption and low levels of physical exercise [3]. Smoking also increases the risk of colorectal cancer in individuals with cancer predisposition syndromes such as Lynch syndrome [3].

### Current colorectal cancer screening methods in Austria

To date, colorectal cancer screening in Austria is opportunistic including the use of colonoscopy or stool-based blood tests.

Currently used stool-based blood tests include guaiac-based fecal occult blood tests (gFOBT), which have been available since the early 1980s or the more recently available fecal immunochemical tests (FIT). If a stool-based blood test is positive, colonoscopy is always indicated as a diagnostic follow-up examination, regardless of the usual screening intervals. The main imaging procedure used for colorectal cancer screening in Austria is colonoscopy. Public health authorities have offered colonoscopy in asymptomatic populations since 2005. The most recent clinical practice guidelines on colonoscopy screening were published by the Austrian Society of Gastroenterology and Hepatology (ÖGGH) in 2016 [4]. For asymptomatic persons, the guidelines recommend colonoscopy screening every 10 years starting at age 50 years up to age 75 years. Other recommendations are based on family or personal history and screening intervals or starting ages vary depending on these factors. Stool-based blood tests are not addressed in the screening recommendations.

### The Austrian National Committee for Cancer Screening (ANCCS)

In January 2021, the Austrian Ministry of Social Affairs, Health, Care and Consumer Protection established the Austrian National Committee for Cancer Screening (ANCCS) as an advisory committee to the Minister of Health. The tasks of the ANCCS are to develop independent evidence-based recommendations on cancer screening and to support the implementation of organized cancer screening programs in Austria. Its members, represented by the co-authors of this publication, are experts in public health, epidemiology, health decision science, oncology, pathology, health economics, law, and ethics, and patients’ or citizens’ representatives. Consultation by the committee members is honorary and occurs free of charge. All members must declare their potential conflicts of interest.

## Objective and target population

The objective of the ANCCS was to provide evidence-based guidance for a nationwide organized colorectal cancer screening program in Austria. The recommendations are directed at asymptomatic adults at an average risk for colorectal cancer (i.e., persons with no prior diagnosis of colorectal cancer, adenomatous polyps, or inflammatory bowel disease and without a personal diagnosis or family history of colorectal cancer, or a genetic disorder that confers an increased lifetime risk of colorectal cancer).

## Methods

The ANCCS is committed to high international recommendation developmental standards as outlined by the US National Academy of Medicine [5]. In general, the methodological framework followed the approach of the US Preventive Services Task Force guidance [6].

### Topic refinement

**Fig. 1:**
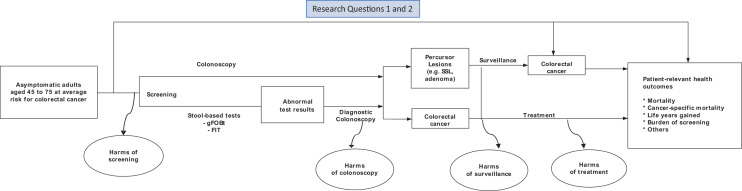
Analytic framework for colorectal cancer screening. *FIT* fecal immunochemical test, *gFOBT* guaiac fecal occult blood test, *SSL* sessile serrated lesion

In an iterative process involving the Ministry of Health and stakeholders, members of the ANCCS developed research questions and refined a population, intervention, comparator and outcomes (PICO) framework for inclusion criteria for studies of interest. A modified Delphi approach was used to select screening interventions of interest for the Austrian context and to determine which outcomes are relevant for informed decision-making about colorectal cancer screening. Figure 1 presents an analytic framework for colorectal cancer screening. Table 1 presents inclusion and exclusion criteria to define the scope of the recommendations. The evidence review was guided by two research questions:

**Table 1:** Inclusion and exclusion criteria

	Inclusion	Exclusion
Screening population	Asymptomatic persons at average risk for colorectal cancer	Symptomatic persons, persons with an increased risk for colorectal cancer
Screening tests	Colonoscopy Fecal immunochemical test (FIT) Guaiac fecal occult blood test (gFOBT)	Capsule endoscopy CT colonography Digital rectal examination Serum tests Sigmoidoscopy Stool DNA tests Urine tests
Screening interval	Different intervals for included screening tests	N/A
Comparator	*Research question 1:* No screening All included screening tests *Research question 2:* All included screening tests	N/A
Outcomes	Incidence of colorectal cancer Colorectal cancer-specific mortality All-cause mortality Serious adverse events leading to hospitalization or death (e.g., perforation, major bleeding, cardiovascular events, other) Proportion of (false) positive test results Remaining life-expectancy (life-years gained) Quality-adjusted remaining life-expectancy (QALYs) Adherence (e.g., participation rates)	Intermediate outcomes (e.g., sensitivity, specificity, number of detected adenomas)

*CT* computed tomography, *DNA* deoxyribonucleic acid, *N/A* not applicable

#### Research question 1

What is the comparative effectiveness of colorectal cancer screening strategies in reducing colorectal cancer incidence and/or mortality?

- Which test or which combination of tests (sequential, parallel) should be used for an organized colorectal cancer screening program?
- For which age groups should an organized colorectal cancer screening program be implemented?
- What is the optimal screening interval for different screening tests?
- Does the effectiveness of screening strategies differ among subgroups (e.g., age, sex, ethnicity)?

#### Research question 2

What are the incremental benefit-harm ratios and incremental benefit-burden ratios for different screening strategies?

### Evidence review

The Austrian National Public Health Institute for Health Promotion, Quality, Planning and Research (Gesundheit Österreich GmbH) conducted an evidence review based on the research questions and the inclusion criteria outlined above [7].

### Assessing the net benefit of the screening intervention

A net benefit exists when the benefits of a preventive intervention outweigh the harms. An important aspect in determining a net benefit is the assessment of the certainty of the available evidence. The certainty of evidence reflects the extent to which the confidence in an estimate of the effect is adequate to support a particular recommendation [8]. To determine the certainty of the evidence, the Austrian National Public Health Institute for Health Promotion, Quality, Planning and Research used the Grading of Recommendations, Assessment, Development and Evaluation (GRADE) approach [9, 10]. When assessing the net benefit of a screening intervention, the ANCCS primarily considered patient-relevant health outcomes, i.e., outcomes that individuals can feel or experience (e.g., cancer-specific mortality, incidence of cancer, major bleeding, perforation, potential psychological harms).

### Development of recommendations

In a structured process, members of the ANCCS considered 1) the magnitude of the net benefit, 2) the certainty of evidence, and 3) the level of intervention acceptability in the target population. The ANCCS did not consider the financial costs or cost-effectiveness of the screening interventions. The ANCCS used the US Preventive Services Task Force recommendation grades, which are summarized in Table 2.

**Table 2:** Recommendation grades and interpretations of the Austrian National Cancer Screening Committee (ANCCS)^a^

Grade	Definition	Implications for practice
A	The ANCCS recommends this service. There is a high certainty of evidence that the net benefit is substantial	Offer or provide this service
B	The ANCCS recommends this service. There is a high or moderate certainty of evidence that the net benefit is at least moderate	Offer or provide this service
C	The ANCCS recommends selectively offering or providing this service to individual patients based on professional judgment and patient preferences. There is at least a moderate certainty of evidence that the net benefit is small	Offer or provide this service for selected patients depending on individual circumstances
D	The ANCCS recommends against the service. There is a moderate or high certainty of evidence that the service has no net benefit or that the harms outweigh the benefits	Discourage use of this service
I Statement	Current evidence is insufficient to assess the balance of the service’s benefits and harms. Evidence is lacking, of poor quality, or conflicting, and the balance of benefits and harms cannot be determined	If the service is offered, individuals should understand the uncertainty about the balance of benefits and harms

^a^Adapted from the US Preventive Services Task Force [6]

### Review of draft recommendations

The Austrian National Public Health Institute for Health Promotion, Quality, Planning and Research organized a meeting with Austrian stakeholders to present and discuss draft recommendations. Comments from the review process were taken into consideration by the ANCCS.

## Recommendation statements

All recommendations apply only to persons without signs or symptoms of colorectal cancer who are at an average risk for colorectal cancer. Based on the evidence review, decision-analytic modeling based on the Austrian data (both presented under “Summary of the supporting evidence”), and stakeholder feedback, the ANCCS developed the following recommendations, which are summarized in Table 3.

1. The ANCCS recommends a quality-assured colorectal cancer screening program with colonoscopy or FIT for individuals aged 45–75 years. In persons 65 years or older, screening decisions should occur on an individual basis in accordance with a person’s overall health, prior screening history, and preferences. Both screening strategies are considered equivalent, and citizens should be able to make an informed decision about their preferred method. Switching between the two screening strategies should be possible (A recommendation, moderate evidence).
2. In persons with unremarkable screening results, the ANCCS recommends that colonoscopy screening should be repeated every 10 years and screening with FIT every 2 years. Persons who have had a colonoscopy screening do not need to be screened with a FIT for another 10 years (A recommendation, moderate strength of evidence).
3. For individuals with pre-existing conditions associated with an increased risk of colorectal cancer (e.g., ulcerative colitis or Crohn’s disease, or in the case of relatives with familial colorectal cancer syndrome), recommendations for colorectal cancer screening differ. Participation in the screening program is possible, but not recommended. These individuals should receive special screening recommendations in specialized centers, according to their diseases, medical history and increased risk. This also applies to individuals already being treated for colorectal cancer. In these cases, an individual decision regarding screening should be made in consultation with the treating physician, according to clinical practice guidelines (good practice statement).
4. The ANCCS recommends accompanying the implementation and ongoing operation of a screening program for colorectal cancer with a public information campaign, systematic data collection, quality assurance, and program evaluation. Furthermore, the ANCCS recommends establishing a strategic steering group and a project steering committee for the implementation of the screening program (taking into account layperson-oriented information and communication) and its documentation, quality assurance, and evaluation (good practice statement).

**Table 3:** Summary of recommendations

Population	Recommendation	Strength of recommendation
Adults aged 45–75 years^a^	The ANCCS recommends the implementation of an organized colorectal cancer screening program for all adults aged 45–75 years	A (moderate evidence)
Adults aged 45–75 years^a^	The ANCCS recommends screening with colonoscopy (every 10 years) or fecal immunochemical test (FIT) every 2 years. Persons who have had a colonoscopy screening do not need to be screened with FIT for the next 10 years	A (moderate evidence)

^a^Persons who do not have signs or symptoms of colorectal cancer and who are at average risk for colorectal cancer

## Summary of the supporting evidence

The ANCCS assessed the net benefits of colorectal cancer screening with colonoscopy or stool-based tests (either gFOBT or FIT). Because of the lack of relevance for the Austrian context, the assessment did not include capsule endoscopy, computed tomography (CT) colonography, digital rectal examinations, serum tests, sigmoidoscopy, stool DNA tests, or urine tests.

### Stool-based tests

A systematic review by Lin et al. identified various studies that confirmed the general efficacy of stool-based tests to reduce colorectal cancer-specific mortality [11]. Based on five randomized controlled trials (RCTs; *N* = 404,396), Lin et al.’s pooled results reported that biennial screening with gFOBT (2–9 rounds of screening) was associated with a reduction in colorectal cancer-specific mortality compared with no screening after 11–30 years (risk ratio, RR 0.78; 95% confidence interval, CI 0.65–0.93 at 30 years) [11].

Likewise, a prospective cohort study (*N* = 5,417,699) evaluating a Taiwanese screening program reported that 1–3 rounds of screening with a biennial FIT led to a lower colorectal cancer mortality than no screening (adjusted RR 0.90; 95% CI 0.84–0.95) [12].

Evidence on the comparative effectiveness of screening with gFOBT and FIT was limited to two RCTs [13, 14] and a prospective cohort study [15]. These studies assessed colorectal cancer detection rates but did not assess cancer-specific mortality. Nevertheless, the results showed consistently higher detection rates for FIT than for gFOBT. The RRs for colorectal cancer detection ranged from 1.84 (95% CI 0.71–4.79) to 2.46 (95% CI 1.82–3.33), favoring FIT over gFOBT [11]. The pooled sensitivity for FIT was 0.74 (95% CI 0.64–0.83) [11], for gFOBT sensitivities ranged from 0.50–0.75 (95% CI 0.09–1.0) in 2 studies [11]. The specificities were similar between FIT and gFOBT [11].

A decision-analytic modeling study for the Austrian context performed by Jahn et al. on behalf of the Austrian Colorectal Cancer Screening Model Group reported that FIT led to more life-years gained with fewer (false) positive tests than gFOBT, regardless of the screening intervals and the start or end screening age [16]. Biennial screening with FIT (from 45–75 years of age) would lead to 436 life-years gained compared with 407 when using gFOBT. At the same time, FIT would lead to 1514 additional colonoscopies compared to 1743 with gFOBT [16].

Apart from the risk of missing cancers or advanced adenomas (false negative results), no serious adverse events occur with noninvasive stool-based tests. Serious adverse events, however, may result from follow-up colonoscopy for abnormal stool-based tests. Therefore, the rate of false positive results, which can lead to unnecessary follow-up colonoscopy, is an important parameter to consider. Because of the similar specificities of gFOBT and FIT, however, false positive findings are likely to occur at similar rates.

Based on the available evidence, the ANCCS recommends the use of FIT as a stool-based test for colorectal cancer screening (A recommendation, moderate evidence).

### Colonoscopy

Based on the review by Lin et al., two prospective observational studies reported a lower incidence of colorectal cancer and colorectal cancer-specific mortality for people who underwent colonoscopy compared with no screening [11]. In one study (*N* = 88,902) the colorectal cancer-specific mortality after 24 years was lower in people who self-reported at least one screening colonoscopy compared with those who had never had a screening colonoscopy (adjusted hazard ratio, HR 0.32; 95% CI 0.24–0.45) [17]. The other study (*N* = 348,025) reported a lower risk for developing colorectal cancer in people aged 70–74 years after 8 years of follow-up [18] (absolute risk reduction −0.42 percentage points; 95% CI −0.24 to −0.63). In people aged 75–79 years, the benefit was smaller and no longer statistically significant. The Nordic-European Initiative on Colorectal Cancer (NordICC) [19], a pragmatic RCT (*N* = 84,585) from Norway, Poland, and Sweden, was published after the ANCCS recommendations meeting. The NordICC reported smaller screening effects than the abovementioned observational studies after 10 years of follow-up but, overall, confirmed the benefits of screening with colonoscopy.

Regarding the harms of colonoscopies, the systematic review by Lin et al. included data of 21 observational studies (*N* = 903,872) to assess serious adverse events. Major bleedings occurred in 17.5 per 10,000 procedures (95% CI 7.6–27.5) and perforations in 5.7 per 10,000 procedures (95% CI 2.8–8.7) [11].

### Comparative effectiveness of stool-based tests and colonoscopy

The systematic review by Lin et al. did not detect any prospective studies that directly compared the screening strategies of stool-based tests and colonoscopy [11]. The best available evidence was an RCT that compared a single colonoscopy or flexible sigmoidoscopy with four rounds of FIT [20]. The colorectal cancer detection rates were similar between participants receiving colonoscopy and those receiving FIT (0.63% vs. 0.77%). Other studies compared the colorectal cancer detection rates of a single colonoscopy with one-time testing with FIT [21–23]. No eligible evidence was available on the screening intervals and start and end age to enable colonoscopy and FIT screening comparisons. Due to the lack of direct evidence regarding the screening intervals and start and end screening age, the ANCCS took modeling studies into consideration.

The UMIT TIROL was tasked with updating a former decision-analytic modeling study for the Austrian context to assess different screening intervals as well as different start and end ages for colorectal cancer screening [16]. Based on the Austrian experience with its current opportunistic colorectal cancer screening, an attendance rate of 29% for colonoscopy and 39% for FIT was used in the base-case analysis of the modeling study. All screening strategies in the model were more beneficial than no screening with respect to life-years gained (range 366–488 life-years gained for 1000 participants from age 40 years to death, depending on the strategy). By lowering the starting age of biennial FIT screening from 50 years to 45 years, 2 additional deaths per 1000 persons screened were prevented (41 years of life gained); however, this change also resulted in 242 additional colonoscopies (6 additional colonoscopies per life-year gained). Likewise, by commencing colonoscopy screening at the age of 45 instead of 50 years (with 3 screening rounds from 45–75 years), 2 additional deaths per 1000 persons were prevented (41 years of life gained). This change, however, resulted in 798 additional colonoscopies (19 additional colonoscopies per life-year gained).

A modeling study by Knudsen et al. simulated 163 different screening strategies in a hypothetical cohort of US adults aged 40 years with no prior cancer diagnosis [24]. Similar to the results from the Austrian study by Jahn et al., the findings showed that commencing screening at age 45 years results in more life-years gained and fewer colorectal cancer cases and deaths than similar strategies with screening initiation at ages 50 or 55 years, albeit with a higher lifetime burden of colonoscopy and non-colonoscopy examinations and a slightly higher lifetime risk of complications [24].

Based on the available evidence, primarily from the decision-analytic modeling study by Jahn et al. reflecting the Austrian healthcare context, the ANCCS recommends a quality-assured colorectal cancer screening program with colonoscopy or FIT for individuals aged 45–75 years (individualized from age 65 years). Both screening strategies are considered equivalent, and citizens should be able to make an informed decision about their preferred method (A recommendation, moderate evidence). The ANCCS recommends colonoscopy and FIT as equivalent screening strategies because the acceptance of colonoscopy in the Austrian population is low (currently about 29%). Offering FIT as a second equivalent strategy can help reach persons who are unwilling to undergo colonoscopy screening.

## Practical implications

Screening programs are useful when a strong evidence base supports their effectiveness [25]. The potential harm, burden, and costs for the healthcare system, and the need for quality assurance must be carefully weighed against the potential benefits of a screening program in terms of a reduction in incidence or mortality.

A first step toward establishing an organized colorectal cancer screening program in Austria was the establishment of a committee (the ANCCS) to develop evidence-based recommendations and report the results to the Minister of Health. Whether an organized colorectal cancer screening program will be implemented in Austria, however, is a political decision. Overall, the screening tests for colorectal cancer meet Wilson and Jungner’s principles [26] for assessing a screening program’s usefulness. An important aspect is “the collection, analysis, and reporting on outcomes to identify false negatives and to improve the effectiveness and cost-effectiveness of the screening program” [25, p. 8]. The performance of the screening tests used as part of the colorectal cancer screening program has already been assessed/reported, and potential outcomes, both benefits and harms, along the entire screening pathway, have been considered including country-specific decision-analytic modeling. An additional cost-effectiveness analysis for a lifelong time horizon would be warranted [27], but an appraisal of Wilson and Jungner’s criteria [26] suggests that a colorectal cancer screening program in Austria would be cost-effective.

Once the political commitment to establish an organized colorectal cancer screening program in Austria has been made, it will be crucial to pilot test the program. Pilot testing represents an important preparatory step before scaling up the program to the national level. Pilot testing to further assess the feasibility, resource use implications, and delivery strategies for an Austrian-wide colorectal cancer screening program should explore how screening performs under varying circumstances. A pilot study, for example, could be set up as a cluster-randomized pragmatic trial, which must be representative of the average national conditions in which the full-scale screening program will be implemented. Additional measures that should be elicited through a pilot study include real-life cost-effectiveness and efficiency, and uptake.

Additional important measures that should be collected during the screening program’s piloting phase include aspects both on the benefits (increasing choices, reducing severity including less invasive treatment, reducing incidence and deaths) as well as harms (overdiagnosis, false negatives or positives, diversion of health resources). New evidence on these aspects can be used to update modeling results and decrease uncertainty. In addition, any pilot of a screening program must be accompanied by a formative evaluation to identify barriers to and facilitators of the program. Decision making should be guided by a set of principles suggested by a World Health Organization (WHO) consultation: respect for dignity and autonomy, nonmaleficence and beneficence, justice and equity, prudence and precaution, and honesty and transparency. These principles require evidence-based information products for citizens and primary healthcare providers on the baseline risks of colorectal cancer, the benefits and harms of different screening modalities, and the burden of screening tests. Such information products can help both individuals and their healthcare providers achieve informed and shared decision-making about the preferred screening approach.

In conclusion, an organized colorectal cancer screening program in Austria is the most effective way to reduce the incidence and mortality of colorectal cancer through the identification and treatment of precancerous stages of colon cancer and the early detection and early treatment of asymptomatic cancers.
