# Correction to: Screening for colorectal cancer

**DOI:** 10.1007/s00508-023-02251-y

**Published:** 2023-08-02

**Authors:** Gerald Gartlehner, Eva Schernhammer, Sigurd F. Lax, Matthias Preusser, Herbert Bachler, Harald Titzer, Maria Kletecka-Pulker, Helga Turnher, Uwe Siebert

**Affiliations:** 1 Department for Evidence-based Medicine and Evaluation, University of Krems, Dr.-Karl-Dorrek-Straße 30, 3500 Krems, Austria; 2 RTI International, Research Triangle Park, NC USA; 3 Center for Public Health, Department of Epidemiology, Medical University of Vienna, Kinderspitalgasse 15, 1090 Vienna, Austria; 4 Pathology, Hospital Graz II, Graz, Austria; 5 Johannes Kepler University, Linz, Austria; 6 Division of Oncology, Department of Medicine I, Medical University of Vienna, Vienna, Austria; 7 Tyrolean College of General Medicine, Medical University of Innsbruck, Innsbruck, Austria; 8 Austrian Society of Hematology and Oncology Nurses, Vienna, Austria; 9 Institute for Ethics and Law in Medicine, University of Vienna, Vienna, Austria; 10 Colorectal Cancer Patient Support Group, Vienna, Austria; 11 UMIT TIROL—University for Health Sciences and Technology, Hall in Tirol, Austria; 12 Harvard T.H. Chan School of Public Health, Boston, USA; 13 Institute for Technology Assessment, Massachusetts General Hospital, Harvard Medical School, Boston, MA USA

**Correction to:**

**Wien Klin Wochenschr 2023**

10.1007/s00508-023-02209-0

In this article the familiy name of “Harald Tietzer” has been misspelled. It should read: Harald Titzer.

The original article has been corrected.

